# Sarcopenia for outcomes in patients undergoing spinal surgery: A protocol for a systematic review and pooled analysis of observational studies

**DOI:** 10.1371/journal.pone.0264268

**Published:** 2022-03-11

**Authors:** Teng Wan, Zhihong Xiao, Xitao Wang, Haifeng Tan, Weiming Guo, Guojun Tang, Liangyuan Chen, Zubing Mei, Qi Cao

**Affiliations:** 1 Department of Spine Surgery, The Second Affiliated Hospital, University of South China, Hengyang, Hunan, China; 2 Departments of Physiology and Biochemistry, Hengyang Medical College, University of South China, Hengyang, Hunan, China; 3 Department of Surgery, Binhai New Area Hospital of Traditional Chinese Medicine, Tianjin, China; 4 Department of Anorectal Surgery, Shuguang Hospital, Shanghai University of Traditional Chinese Medicine, Shanghai, China; 5 Anorectal Disease Institute of Shuguang Hospital, Shanghai, China; Stanford University School of Medicine, UNITED STATES

## Abstract

**Background:**

Sarcopenia is a progressive age-related skeletal muscle disorder characterized by decreased muscle mass and loss of muscle function. Recent studies have shown that sarcopenia is able to predict a variety of clinical outcomes after spinal surgery. Controversy still exists among previous reports in terms of the definition and measurement of sarcopenia, these findings are heterogeneous so far. Therefore, the aim of the current study is to assess the up-to-date evidence of sarcopenia for postoperative outcomes among people undergoing spinal surgery.

**Methods and analysis:**

This protocol was carried out based on the preferred reporting items for systematic review and meta-analysis protocols (PRISMA-P) statement. It has been pre-registered in PROSPERO with the registration number of CRD42021260459. Three databases (including Pubmed, EMBASE, and Cochrane Library) will be searched from inception through May 10, 2021 to determine related cohort studies examining sarcopenia on multidimensional outcomes in patients undergoing spinal surgery. Major outcomes will be involved including mortality, morbidity, length of stay, postoperative complications or adverse events. DerSimonian & Laird random-effects meta-analysis will be used to calculate pooled odds ratio (OR) for binary data and pooled weighted mean differences (WMDs) or standardized mean differences (SMDs) for continuous data. The Newcastle-Ottawa Scale (NOS) will be used to assess the risk of bias of included studies. Narrative synthesis will be carried out if a pooled analysis is not possible.

**Ethics and dissemination:**

Ethical approval is not required for this study as the data involved are from the published literatures. We intend to disseminate or share the results of the study in a peer-reviewed journal or at relevant conferences.

**PROSPERO registration number:**

CRD42021260459.

## Introduction

Sarcopenia is a progressive age-related skeletal muscle disorder characterized by decreased muscle mass and loss of muscle function. In addition, activity limitation, muscle anabolism resistance, lipid cytotoxicity and inflammation are all factors that contribute to this primary condition [1]. In addition, another condition, also known as secondary sarcopenia, is characterized by a decline in muscle mass because of the decreased level of activity, malnutrition and organ failure caused by advanced diseases such as metastatic tumors [2]. The condition of sarcopenia has many negative effects on patients, including falls, dysfunction, osteoporosis, frailty and increased mortality [3]. In recent years, the incidence of spinal injury, scoliosis, intervertebral disc herniation, a variety of spinal degenerative diseases and malignant spinal metastases has gradually increased. Spinal surgery is a common treatment for most spinal diseases. However, the high incidence of postoperative complications and adverse events following spinal surgery has not been well managed and resolved [4]. Further analysis and evaluation of the risk factors affecting the outcomes of spinal surgery and their predictive value for postoperative outcomes are needed to provide a reliable indicator for clinical prediction of adverse outcomes after spinal surgery.

Recent studies have shown that sarcopenia is able to predict a variety of clinical outcomes after spinal surgery. Toyoda et al. found that different stages of sarcopenia, including low muscle mass, low muscle strength, and low body performance, contributed to the increased risk of poor clinical outcome of lumbar decompression surgery [5]. Another study showed that in complex thoracolumbar revision surgery, sarcopenia could predict postoperative readmission, reoperation, in-hospital mortality, and other adverse events [6]. In patients with spinal metastases, sarcopenia predicted postoperative survival, early mortality, and adverse events [7–10]. It has also been reported that sarcopenia predicts the morbidity after lumbar surgery [11]. However, several other studies have come to the opposite conclusion, which found that in the elderly population of non-complex lumbar surgery due to degenerative changes, sarcopenia could not predict adverse events, in-hospital mortality, length of stay and postoperative complications [12, 13]. In addition, it has been reported that in patients with lumbar fusion, there is no significant difference in postoperative clinical outcomes between sarcopenia and non-sarcopenia groups [14].

As some controversy still exists among previous conflicting reports in terms of the definition and measurement of sarcopenia, these findings are obviously heterogeneous so far [15]. At the same time, different population characteristics, such as gender, age, race, type of disease, different forms of surgery (e.g. minimally invasive, open, and different surgical sites) may also be factors affecting the postoperative outcome of sarcopenia [5, 9]. In order to comprehensively determine the predictive value of sarcopenia on multiple outcomes of patients following spinal surgery, we will carry out a systematically review with pooled analysis based on the published cohort studies. Moreover, we also try to justify if sarcopenia is associated with poor surgical outcomes and adverse events for patients undergoing spinal surgery.

## Methods and analysis

### Protocol registration

This study will be performed based on Preferred Reporting Items for Systematic Review and Meta-Analyses Protocol (PRISMA-P) guideline [16] (S1 Table). The protocol was registered on the website of https://www.crd.york.ac.uk/prospero/ on June 13, 2021 (Registration number: CRD42021260459). We plan to conduct this study between May 2021 and December 2021.

### Data sources and search strategies

The literature search was conducted using PubMed, Embase and Cochrane Library databases from inception to May 10, 2021 to determine eligible observational studies on the impact of sarcopenia on postoperative outcomes in patients undergoing spinal surgery. The search will apply Medical Subject Headings and free-text words related to the following three elements: sarcopenia, spinal surgery, and clinical outcomes (S2 Table). We will not include grey literature such as unpublished conference proceedings or abstracts from relevant conference meetings due to potential high risk of bias. In addition, we will also manually screen the reference list of included studies to identify additional studies that meet the inclusion criteria, with the aim to minimize the missing literatures. If the related data provided in the original articles are unclear or insufficient, we will contact the corresponding authors to obtain detailed information.

### Study selection and eligibility criteria

Relevant observational studies that describe the clinical outcomes in sarcopenia patients undergoing spinal surgery are identified by T.W. and Z.X. The titles, abstracts and full texts (if necessary) will be reviewed to determine eligibility of the study on the basis of inclusion and exclusion criteria. Any discrepancy will be resolved through discussion; otherwise, a senior author (Z.M.) will be consulted.

The inclusion criteria of this study are summarised using the PECOS (participants, exposure, comparison, outcome, study design) framework according to Cochrane handbook [17].

### Participants

Surgically treated patients (over 18 years of age) with an established diagnosis of spine diseases including spinal injury, scoliosis, intervertebral disc herniation, a variety of spinal degenerative diseases or malignant spinal metastases based on accepted criteria (such as clinical symptoms and imaging examinations) will be included.

### Exposure

Sarcopenia is the primary exposure of interest. Therefore, studies will be included in the systematic review if their exposed participants have a confirmed diagnosis of sarcopenia.

Studies in which the exposure status (both primary and secondary sarcopenia) is mainly determined according to the European Working Group on Sarcopenia in Older People (EWGSOP) (2010), EWGSOP2 (2018), the Foundation for the National Institutes of Health Sarcopenia Project (FNIH) (2014) and Asian Working Group for Sarcopenia (AWGS) algorithm [18, 19]. Sarcopenia can also be measured by low muscle mass, low skeletal muscle strength, and poor physical performance. A non-sarcopenia cohort is defined as the non-exposure (comparison) group.

### Outcomes

Studies will be included in this systematic review if one of the outcomes will be involved (1) major adverse outcomes of spinal surgery; (2) length of stay; (3) adverse events.

Major adverse outcomes of spinal surgery will include:

- Lower back pain, lower extremity pain, and lower extremity numbness, assessed using Japanese Orthopaedic Association (JOA) and visual analogue scale (VAS) scores [20].
- Postoperative complications, commonly including postoperative visual loss, peripheral nerve injury, skin damage, postoperative nausea and vomiting and deep vein thrombosis collected according to Clavien-Dindosystem [21].
- Morbidity rate, which is calculated and assessed by the Charlson Comorbidity Index [22]
- Mortality, including in-hospital mortality, 30-day postoperative mortality, 90-day postoperative mortality and overall mortality.

Length of stay refers to duration between admission and discharging.

Adverse events include anemia requiring blood transfusion, cardiac arrest, septicemia, wound complications (rupture, infection), confusion, intraoperative dural tear, acute renal injury, pneumonia, urinary tract infection, urinary retention, epidural hematoma, deep venous thrombosis, etc. [8]. Adverse events will be collected using spinal event severity system version 2 (SAV2.0) [23].

### Data extraction

Two reviewers will independently extract the date using a piloted standardized data extraction form. Any discrepancies will be resolved through discussion and, if necessary, the corresponding authors of the original text will be contacted to obtain correct and complete information. The following information will be extracted from each study:

- First author
- Year of publication
- Study design
- Study region or country
- Observation period
- Sample size of the involved participants
- Sex ratio
- Preoperative diagnosis
- Comorbidity
- Surgical methods
- Surgical site
- Surgical acuity
- Definition and measurement of sarcopenia
- Outcomes with their definitions
- Follow-up period
- Adjusted variables

### Methodological quality assessment

Methodological quality (risk of bias) will be assessed using Newcastle-Ottawa Scale (NOS) tool [24]. Any discrepancies will be resolved through consensus. For each eligible observational study, the following domains will be assessed: the representativeness of the exposed cohort, the selection of the control (non-exposed) cohort, the determination of exposure factors, whether the outcome events occur before the start of follow-up, the comparability of the cohort, the assessment of the outcome events that occurred, the duration of follow-up, and report on the levels of loss to follow-up [25]. A score of NOS 7 or less corresponds to low study quality (a high risk of bias), and a score greater than 7 corresponds to high study quality (a low risk of bias).

### Data synthesis

Stata statistical software (version 15.0; STATA, College Station, TX) will be used for all meta-analysis. We will abstract directly or calculate odds radio (OR) and 95% CI as the effect measure for dichotomous variables. While for continuous outcome variables, weighted mean difference (WMD) (if all the studies use the same measurement tool and the same unit) or standardized mean difference (SMD) (if studies use various measurement tools or different units) will be abstracted along with their corresponding 95% CIs for meta-analysis. Dichotomous outcome variables will be pooled using the Mantel Haenszel method and continuous outcome variables will be pooled using the inverse variance method [26]. Between-study heterogeneity will be evaluated by Cochrane Q test with a P value more than 0.10 indicating no heterogeneity or slight heterogeneity, while P more than 0.10 implying significant heterogeneity. Besides, heterogeneity will also be assessed by the I^2^ statistics with I^2^ values more than 50% considering significant heterogeneity [27, 28]. A summary of exposure effect estimates will be calculated using the more conservative approach of a random effects model. In addition, sensitivity analysis will be performed to test the robustness of pooled estimates. Multiple subgroup analyses will also be performed to further explore the source of between-study heterogeneity stratified by disease type (degenerative spinal disease vs. traumatic spinal injury vs. spinal metastatic disease), sarcopenia definition (EWGSOP vs. AWGS) and other variables. When a sufficient number of studies (≥10 studies) are included for meta-analysis, publication bias will be assessed by visual inspection of the funnel plot symmetry and further by Egger’s linear regression test [29]. We will only provide a narrative description instead of meta-analysing the study outcome data if significant heterogeneity exists. If a publication bias is suggested, we will utilize the Duvall and Tweedle trim-and-fill model to adjust the effect estimates [30].

## Discussion

The impact of sarcopenia on outcomes in patients undergoing spinal surgery is still controversial. The predictive value of sarcopenia on outcomes in those patients has been challenged by the highly heterogeneous results due to inconsistences in definition, measurement tools, and the wide variability in surgical methods. In this study, we will provide a general overview of summary review of sarcopenia on clinical outcomes of those people by incorporating evidence from published observational studies.

Several strengths should be addressed for our study. Firstly, this will be the first systematic review to comprehensively evaluate the up-to-date evidence to clarify the impact of sarcopenia on multidimensional outcomes in patients undergoing spinal surgery. Secondly, this study will synthesize a board range of evidence on outcomes of patients undergoing spinal surgery from all the available evidence, providing sufficient objective evidence for surgeons or clinicians to support the clinical decision. In fact, our study involves issues of widespread concern to spinal surgery patients, such as lower back pain, lower extremity pain, and lower extremity numbness, postoperative complications, morbidity, mortality and length of stay. Thirdly, multiple sensitivity analyses will be performed to test the robustness of the findings of each outcome to make the results more reliable. Finally, the search strategy and study protocol strictly adhere to the PRISMA statement for the conduct and report of systematic reviews and meta-analysis.

The findings of this study will be disseminated or shared in a peer-reviewed journal or at relevant conferences, and we believe that the results will benefit spine surgeons, spine surgical patients and policy-makers.

## Supporting information

S1 Table PRISMA-P checklist. (DOC)

S2 Table Search strategies for three databases. (DOCX)

## Abbreviations

AWGS: Asian Working Group for Sarcopenia
EWGSOP: the European Working Group on Sarcopenia in Older People
FNIH: the Foundation for the National Institutes of Health Sarcopenia Project
JOA: Japanese Orthopaedic Association PRISMA-P
NOS: Newcastle-Ottawa Scale
VAS: visual analogue scale
OR: odds ratio
Preferred: Reporting Items for Systematic Review and Meta-Analysis Protocols
SMD: standardized mean differences
WMD: weighted mean differences
