# Supplementary material for: Sarcopenia for outcomes in patients undergoing spinal surgery: A protocol for a systematic review and pooled analysis of observational studies
Source: PLoS One. 2022 Mar 11;17(3):e0264268. doi: 10.1371/journal.pone.0264268 (PMC8916656; doi:10.1371/journal.pone.0264268)
Supplement: S2 Table — (DOCX) [file pone.0264268.s002.docx]

**S2 Table--Search strategies**

**Text S1 Search strategy**

**Database: Medline from inception to Present> (Search date: May 10, 2021)**

**Search Strategy:**

--------------------------------------------------------------------------------

***Sarcopenia terms:***

1. "Sarcopenia"[Mesh]
2. "Muscle Strength"[Mesh]
3. "Physical Fitness"[Mesh]
4. "Geriatric Assessment"[Mesh]
5. (Physical performance or Frail* or Geriatric Assessment or Physical function or Muscle strength or Muscle function or Hand grip strength or Gait speed or Walking speed or Fitness or Physical fitness or Body composition or Sarcopeni* or Cachexia or Skeletal muscle or Muscle mass or Skeletal muscle index)[Title/Abstract]
6. 1-5/or

***Spine surgery terms:***

1. "Spinal Diseases"[Mesh]
2. "Scoliosis"[Mesh]
3. "Spinal Fusion"[Mesh]
4. (spine* or spinal or lumbar or thoraco* or thoracic or scolios*)[Title/Abstract]
5. 7-10/or

***Outcome terms:***

1. "Mortality"[Mesh]
2. "Length of Stay"[Mesh]
3. "Morbidity"[Mesh]
4. "Postoperative Complications"[Mesh]
5. "complications" [Subheading]
6. (Morbidit* or length of stay or LOS or discharge disposition or Complication* or Adverse event* or Clavien-Dindo)[Title/Abstract]
7. 12-17/or

***Final search results: Combining Sarcopenia and Spine surgery and Outcome:***

19 6 and 11 and 18

**Text S2 Search strategy**

**Database: EMBASE (Search date: May 10, 2021)**

**Search Strategy:**

--------------------------------------------------------------------------------

***Sarcopenia terms:***

1 'geriatric assessment'/exp

2 'fitness'/exp

3 'muscle strength'/exp

4 'sarcopenia'/exp

5 (‘Physical performance’ or Frail* or ‘Geriatric Assessment’ or ‘Physical function’ or ‘Muscle strength’ or ‘Muscle function’ or ‘Hand grip strength’ or ‘Gait speed’ or ‘Walking speed’ or Fitness or ‘Physical fitness’ or ‘Body composition’ or Sarcopeni* or Cachexia or ‘Skeletal muscle’ or ‘Muscle mass’ or ‘Skeletal muscle index’) :ab,ti

1. 1-5/or

***Spine surgery terms:***

7 'spine fusion'/exp

8 'scoliosis'/exp

9 'spine disease'/exp

1. (spine* or spinal or lumbar or thoraco* or thoracic or scolios*) :ab,ti

11 7-10/or

***Outcome terms:***

12 'complication'/exp

13 'postoperative complication'/exp

14 'morbidity'/exp

15 'length of stay'/exp

16 'mortality'/exp

17 (Morbidit* or ‘length of stay’ or LOS or ‘discharge disposition’ or Complication* or ‘Adverse event’ or Clavien-Dindo) :ab,ti

1. 12-17/or

***Final search results: Combining Sarcopenia and Spine surgery and Outcome:***

18 6 and 11 and 19

**Text S3 Search strategy**

**Database: Cochrane Library from inception to Present> (Search date: May 10, 2021)**

**Search Strategy:**

--------------------------------------------------------------------------------

***Sarcopenia terms:***

#1 MeSH descriptor: [Sarcopenia] explode all trees

#2 MeSH descriptor: [Muscle Strength] explode all trees

#3 MeSH descriptor: [Physical Fitness] explode all trees

#4 MeSH descriptor: [Geriatric Assessment] explode all trees

#5 ((Physical performance or Frail* or Geriatric Assessment or Physical function or Muscle strength or Muscle function or Hand grip strength or Gait speed or Walking speed or Fitness or Physical fitness or Body composition or Sarcopeni* or Cachexia or Skeletal muscle or Muscle mass or Skeletal muscle index)):ti,ab,kw (Word variations have been searched)

#6 #1 or #2 or # 3 or #4 or #5

***Spine surgery terms:***

#7 MeSH descriptor: [Spinal Diseases] explode all trees

#8 MeSH descriptor: [Scoliosis] explode all trees

#9 MeSH descriptor: [Spinal Fusion] explode all trees

#10 ((spine* or spinal or lumbar or thoraco* or thoracic or scolios*)):ti,ab,kw (Word variations have been searched)

#11 #7 or #8 or #9 or #10

***Outcome terms:***

#12 MeSH descriptor: [Mortality] explode all trees

#13 MeSH descriptor: [Length of Stay] explode all trees

#14 MeSH descriptor: [Morbidity] explode all trees

#15 MeSH descriptor: [Postoperative Complications] explode all trees

#16 ((Morbidit* or ‘length of stay’ or LOS or ‘discharge disposition’ or Complication* or ‘Adverse event’ or Clavien-Dindo)):ti,ab,kw (Word variations have been searched)

#17 #12 or #13 or #14 or #15 or #16

***Final search results: Combining Sarcopenia and Spine surgery and Outcome:***

#18 #6 and #11 and #17
